# Historical Factors Associated With Past Environments Influence the Biogeography of Thermophilic Endospores in Arctic Marine Sediments

**DOI:** 10.3389/fmicb.2019.00245

**Published:** 2019-02-28

**Authors:** China A. Hanson, Albert L. Müller, Alexander Loy, Clelia Dona, Ramona Appel, Bo Barker Jørgensen, Casey R. J. Hubert

**Affiliations:** ^1^School of Civil Engineering and Geosciences, Newcastle University, Newcastle upon Tyne, United Kingdom; ^2^Division of Microbial Ecology, Department of Microbiology and Ecosystem Science, University of Vienna, Vienna, Austria; ^3^Austrian Polar Research Institute, Vienna, Austria; ^4^Department of Microbiology, Max Planck Institute for Marine Microbiology, Bremen, Germany; ^5^Center for Geomicrobiology, Aarhus University, Aarhus, Denmark; ^6^Geomicrobiology Group, Department of Biological Sciences, University of Calgary, Calgary, AB, Canada

**Keywords:** biogeography, thermophile, endospore, marine sediment, *Desulfotomaculum*, sulfate-reducing bacteria, dispersal

## Abstract

Selection by the local, contemporary environment plays a prominent role in shaping the biogeography of microbes. However, the importance of historical factors in microbial biogeography is more debatable. Historical factors include past ecological and evolutionary circumstances that may have influenced present-day microbial diversity, such as dispersal and past environmental conditions. Diverse thermophilic sulfate-reducing *Desulfotomaculum* are present as dormant endospores in marine sediments worldwide where temperatures are too low to support their growth. Therefore, they are dispersed to here from elsewhere, presumably a hot, anoxic habitat. While dispersal through ocean currents must influence their distribution in cold marine sediments, it is not clear whether even earlier historical factors, related to the source habitat where these organisms were once active, also have an effect. We investigated whether these historical factors may have influenced the diversity and distribution of thermophilic endospores by comparing their diversity in 10 Arctic fjord surface sediments. Although community composition varied spatially, clear biogeographic patterns were only evident at a high level of taxonomic resolution (>97% sequence similarity of the 16S rRNA gene) achieved with oligotyping. In particular, the diversity and distribution of oligotypes differed for the two most prominent OTUs (defined using a standard 97% similarity cutoff). One OTU was dominated by a single ubiquitous oligotype, while the other OTU consisted of ten more spatially localized oligotypes that decreased in compositional similarity with geographic distance. These patterns are consistent with differences in historical factors that occurred when and where the taxa were once active, prior to sporulation. Further, the influence of history on biogeographic patterns was only revealed by analyzing microdiversity within OTUs, suggesting that populations within standard OTU-level groupings do not necessarily share a common ecological and evolutionary history.

## Introduction

Some microbes have globally widespread distributions while others do not; yet pinpointing the mechanisms responsible for these biogeographic patterns remains challenging (Hanson et al., 2012). A classic perspective of biogeography divides the underlying mechanisms into two main categories: those that are driven by the contemporary, local environment versus those that rely on circumstances that have happened in the past (Martiny et al., 2006). These historical factors include all ecological and evolutionary events that previously influenced (either long ago or relatively recently) a present-day population or community, such as dispersal and past environmental conditions. The role of historical factors in shaping the diversity and biogeography of microbes has been hotly debated for decades because – it is contended – microbes’ enormous potential for rapid and widespread dispersal should erase any signature of past events. However, much recent evidence now suggests that at least one historical factor, dispersal limitation, indeed shapes the biogeography of many microbes (reviewed in Martiny et al., 2006; Hanson et al., 2012).

The degree to which current microbial biogeographic distributions are shaped by historical factors other than dispersal limitation remains largely untested, especially for marine microbes. Thermophilic, fermentative and sulfate-reducing endospore-forming bacteria within the phylum Firmicutes, including members of the genus *Desulfotomaculum*, are useful models for studying the role of historical factors in marine microbial biogeography. These thermophiles are abundant in cold marine sediments as dormant endospores (Hubert et al., 2009; de Rezende et al., 2013; Volpi et al., 2017) and have been detected in marine sediments worldwide (Müller et al., 2014). Because the ambient temperature in sediments and the overlying water column are far too low to support their activity (Hubert et al., 2009, 2010; de Rezende et al., 2013), endospores of thermophiles in cold sediments must be derived from a different ecosystem or location (i.e., they are not autochthonously derived). Further, they remain as dormant endospores and do not undergo endogenous growth in these sediments and are therefore not subject to local selection by environmental factors. Thus, contemporary, local environmental factors have effectively no influence on the distribution of thermophilic spores in cold sediments. Instead, their distribution and diversity is the result of historical factors alone.

Several studies have specifically investigated dispersal of thermophilic endospores by characterizing their biogeographic distribution in marine sediments at regional scales (de Rezende et al., 2013; Chakraborty et al., 2018) and global scales (Müller et al., 2014) as well as in estuarine systems (Bell et al., 2018). These studies have provided growing evidence in support of the hypothesis that many thermophilic endospores are derived from fluid flow expelled from marine deep biosphere ecosystems such as mid-ocean ridge venting systems and deeply buried hydrocarbon reservoirs, and are subsequently deposited to marine sediments via passive dispersal. Many thermophilic endospores identified in these studies are phylogenetically related to other organisms found at hydrothermal vents and/or hydrocarbon reservoirs (Hubert et al., 2009, 2010; Nielsen et al., 2017; Chakraborty et al., 2018). Additionally, the diversity of thermophilic endospores in a single cold sediment can be considerable (de Rezende et al., 2013; Müller et al., 2014; Chakraborty et al., 2018) and may be explained by different taxa originating from different warm sources. These biogeographic studies also show that, despite being well-equipped for long-distance dispersal as spores, dispersal limitation influences the biogeography of some thermophilic endospores, while others are more cosmopolitan in distribution (Müller et al., 2014; Bell et al., 2018; Chakraborty et al., 2018). For example, Müller et al. (2014) detected 146 unique phylotypes (i.e., OTUs with ≥97% 16S rRNA sequence identity) in a global survey of over 80 sediments, with some phylotypes present across many geographically distant locations and others detected in only a few samples. Moreover, similarity in the composition of thermophilic endospore communities significantly decreased with increasing geographic distance (Müller et al., 2014), a relationship that, in the absence of environmental selection, can only be caused by historical factors (Hanson et al., 2012), namely, in this case, dispersal limitation. For thermophilic endospores, we consider this dispersal to be inclusive of dissemination from a warm, allochthonous source environment followed by passive transport in oceanic currents and finally deposition to the seafloor via sedimentation.

But do circumstances prior to this dispersal process, specifically the biological history of thermophilic endospore-forming bacteria in the warm habitat(s) from which they are derived, influence their later distribution and diversity in cold sediments? In this case, history includes two components, which we refer to collectively as “historical factors”: (1) the ecological (both deterministic and stochastic) and/or evolutionary events associated with the former warm source habitat where thermophilic endospore forming populations were once active and (2) the location of those habitats, assuming they are fixed. These factors will have shaped the abundance, community composition, and genetic diversity of active microbes in those locations, which likely include warm habitats in the geosphere (Hubert et al., 2009). When endogenous microbes are expelled into the cold ocean as dormant spores, historical factors associated with their source habitat may therefore leave an imprint on their eventual distribution and diversity, e.g., in cold seabed surface sediments. Some examples of past ecological and evolutionary mechanisms that shape microbial diversity include but are not limited to: physical connectivity or habitat isolation that influenced immigration and gene flow (past dispersal), stochastic demographic changes (past ecological drift), and adaptation to environmental conditions (past selection) (Hanson et al., 2012).

The west coast of Spitsbergen, Svalbard in the Arctic Ocean is an ideal location to address the role of historical factors in shaping the distribution of thermophilic endospores for several reasons. First, the Arctic Ocean north of Iceland is considered a distinct biogeographic province (Longhurst, 1998; Costello et al., 2017), inclusive of Arctic surface water (Costello et al., 2017), deep waters (German et al., 2011; Costello et al., 2017), and its mid-ocean ridge system containing hydrothermal vents (Tyler and Young, 2003; German et al., 2011). A biogeographic province is an area particularly reflective of historical factors due to geological or physical forces and/or dispersal barriers that separate it from other regions (Martiny et al., 2006; Takacs-Vesbach et al., 2008), and for macro-organisms, is often distinguished by endemic taxa (Costello et al., 2017). Indeed, the Arctic is relatively more isolated from global ocean circulation relative to other oceans because of its many bordering large land masses and resulting shallow sills that restrict movement of deep waters between the Arctic and its neighboring oceans (German et al., 2011). Secondly, the west coast of Svalbard is in close proximity to potential warm deep biosphere ecosystems from which thermophilic endospores are likely derived including mid-ocean ridge spreading centers and buried petroleum deposits (Gautier et al., 2009; Pedersen et al., 2010; Jaeschke et al., 2014). The Arctic Mid Ocean Ridge system (AMOR) lies approximately 200 km due west of Spitsbergen and the Gakkel Ridge lies approximately 650 km due north of Spitsbergen; both of these are hydrothermally active with plumes and vent fields (German et al., 2011; Pedersen and Bjerkgård, 2016). In addition, the region is a well-known repository of both explored and unexplored petroleum reservoirs, with the western portion of the Svalbard shelf estimated to contain up to 1 billion barrels of undiscovered oil and 6 trillion cubic feet of undiscovered gas (Gautier et al., 2009). Thus, within this biogeographic province at the small scale of western Svalbard, observed biogeographic patterns for thermophilic endospores should highlight the effect of historical factors associated with one or more nearby warm source environments.

Therefore, the aim of this study was to test whether historical factors associated with a former habitat shape the biogeography of themophilic endospores in sediments near Svalbard. To do this, we characterized their diversity and distribution in ten sampling stations along the west coast of Svalbard using a heated enrichment approach. Diversity was assessed by analysis of organic acid resource use at the whole community level in sediment enrichment incubations and by 16S rRNA gene sequence analysis of the enriched communities. We hypothesized that enriched thermophilic endospore taxa would differ in their presence-absence distribution across our study area (16–240 km). If so, then a comparison of distribution patterns may reveal differences in the historical factors influencing those taxa. Our aim was not to identify the exact location or type of source environment(s), but rather to speculate on the nature of past ecological and evolutionary pressures and the environmental scenarios under which they could have occurred. Further, as past ecological and evolutionary pressures would have direct effects at the population-level, we hypothesized that the influence of historical factors would be relatively more apparent at higher levels of taxonomic resolution (i.e., population-level) (Berry et al., 2012; Hanson et al., 2012; Buttigieg and Ramette, 2014; Eren et al., 2014; Jones et al., 2016; Chase and Martiny, 2018). In particular, we expected biogeographic patterns to be weakest when diversity was defined using resource-use at the community level and greatest when defined using genetic “microdiversity” within 16 rRNA genes at the >97% sequence identity level.

## Materials and Methods

### Sediment Samples and Locations

**FIGURE 1:**
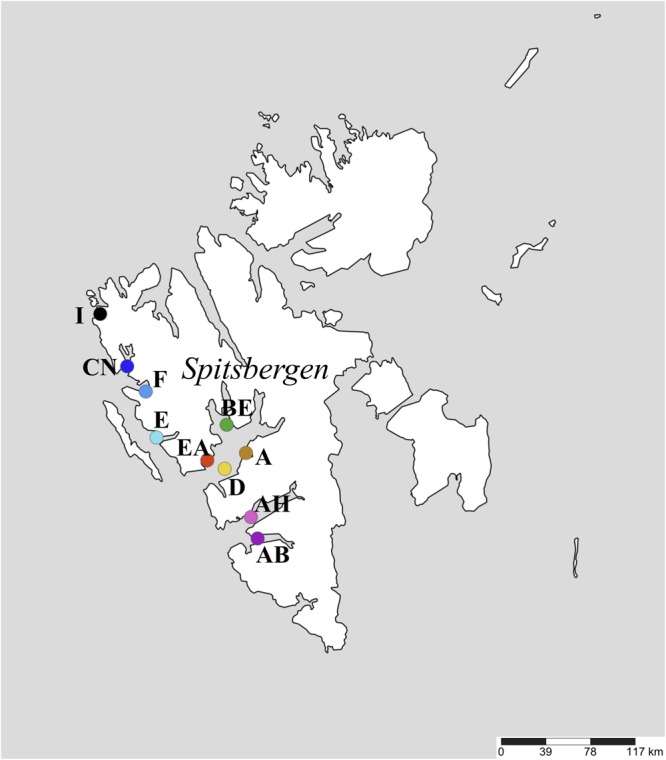
Map showing the ten sediment sampling stations along the west coast of Spitsbergen, Svalbard.

Marine surface sediments (3–9 cm depth) were collected with a Haps corer [a single coring cylinder with top valve supported by a frame (Kanneworff and Nicolaisen, 1983)], aboard R/V *Farm* during the summer of 2006 or 2007 from 10 stations within 7 distinct fjords along the west coast of Spitsbergen, Svalbard (Figure 1 and Table 1). Surface sediments were chosen here because they represent recent deposition via sedimentation within the past 100 years (Hubert et al., 2009) and thus loss of thermophilic endospore viability over time (de Rezende et al., 2013; Volpi et al., 2017) is expected to have minimal influence on their abundance and diversity in surface sediments. Sediments were sealed in gas-tight plastic bags and stored at 4°C. For all stations, the *in situ* surface sediment temperature range was -2 to +7°C. Geodesic distance between stations ranged between 16 and 240 km. A map of stations (Figure 1) was produced using SimpleMappr (Shorthouse, 2010).

**Table 1:** Sediment sampling stations, geographic coordinates, and water depth.

Station ID	Name	Latitude	Longitude	Water depth (m)
A	Adventfjorden	78°15.51′N	15°30.53′E	69
AB	Van Keulenfjorden	77°35.19′N	15°05.24′E	100
AH	Van Mijenfjorden	77°45.75′N	15°03.23′E	116
BE	Nordfjorden	78°30.73′N	15°04.48′E	192
CN	Krossfjorden	79°08.40′N	11°44.18′E	334
D	Isfjorden	78°10.91′N	14°34.12′E	243
E	West of Stor Jonsfjorden	78°32.71′N	12°17.70′E	168
EA	Ymerbukta	78°16.80′N	14°00.31′E	0 (intertidal)
F	Kongsfjorden	78°54.97′N	12°16.04′E	109
I	Magdalenefjorden	79°35.05′N	11°03.59′E	126

### Sediment Slurry Preparation and Incubations at 50°C

Sediment slurries were prepared for each of the 10 stations separately under a constant flow of N_2_ by diluting sediment in a 1:2 (w/w) ratio with artificial seawater medium (Widdel and Bak, 1992) and sulfate concentration adjusted to 20 mM. To evenly enrich, detect, and assess the diversity of sulfate-reducing bacteria (SRB) across the different sediments, a combined pool of typical SRB substrates acetate, butyrate, ethanol, formate, lactate, propionate and succinate were also added to all incubations such that each substrate was provided to a final concentration of 1 mM (Hubert et al., 2010; de Rezende et al., 2013). Slurries were homogenized and dispensed into autoclaved serum bottles under a flow of N_2_ and then sealed with stoppers. These master slurries were divided into two identical portions: one was spiked with radioactive sulfate tracer (^35^S-sulfate) for determination of sulfate reduction, and the other was not spiked but used for organic acid analysis and DNA extraction (described below). To enrich for thermophilic SRB present in the samples as endospores, slurries were pasteurized at 80°C for 1 h, sub-sampled for an initial time-zero sample, then incubated at 50°C for 253–277 h. Subsamples (1–2 ml aliquots) were removed from the slurries every 12–24 h using N_2_-flushed syringes, and analyzed as described below. An incubation temperature of 50°C was chosen because it is in the range of thermophilic growth previously observed in Svalbard sediments (45–64°C) (Hubert et al., 2009, 2010), yields maximal sulfate reduction rates and SRB diversity (Hubert et al., 2009; de Rezende et al., 2013), and to maintain consistency with previous studies (e.g., Müller et al., 2014). This heated incubation approach has several advantages. First, the approach has previously been shown to enrich for a wide diversity of thermophilic spore-forming *Firmicutes* (Müller et al., 2014; Chakraborty et al., 2018), including those that are phylogenetically related to known spore-forming *Desulfotomaculum* spp. (Tardy-Jacquenod et al., 1998; Vandieken et al., 2006; de Rezende et al., 2013). The enrichment is also necessary in order to effectively target only those cells which are present as endospores because it (a) kills vegetative cells, allowing endospores to be distinguished (Müller et al., 2014) and (b) induces germination of endospores, thus circumventing the problem of endospores being missed in direct sequencing libraries due to their resistance to commonly used DNA extraction methods (Wunderlin et al., 2013; de Rezende et al., 2017). The incubations do not necessarily elicit germination and growth of all thermophilic endospore taxa present in the samples, but are effective at enriching communities that are comparable across samples by employing equivalent incubation conditions and substrate amendments (Müller et al., 2014).

### Organic Acid Concentrations and Sulfate Reduction

Concentrations of low-molecular-weight organic acids in filtered subsamples were determined with a Sykam HPLC system equipped with an anion neutral pre-column (4 mm × 20 mm; Sykam GmbH) and an Aminex HPX-87H separation column (300 mm × 7.8 mm; Bio-Rad) at 60°C. The eluent was 5 mM H_2_SO_4_ prepared using HPLC grade water, and the flow rate was 0.6 ml min^-1^. Succinate, lactate, formate, acetate, propionate, and butyrate were quantified using a UV detector (210 nm) and a refractive index detector using single injections (one measurement per sample per time point).

The extent of sulfate reduction at each sampling time point was determined in parallel sediment slurries that contained ca. 100 kBq ml^-1^ of ^35^S-sulfate. Subsamples were fixed in 20% w/v zinc acetate and frozen until determination of sulfate reduction by cold chromium distillation of reduced sulfur compounds as described elsewhere (Kallmeyer et al., 2004). The fraction of added ^35^S in the reduced sulfur pool was used to determine the proportion of sulfate reduced to sulfide, and is reported here as the extent of sulfate reduction. These proportions were calculated using sulfate concentrations in the supernatant that were measured following 100× dilution via non-suppressed anion exchange chromatography (Ferdelman et al., 1997).

### Sequencing and OTU Clustering

We focused on the genus *Desulfotomaculum*, as previous work indicated that sulfate reduction within similar sediment incubations was due to the growth of thermophilic members of this specific genus (Hubert et al., 2009, 2010; Müller et al., 2014). Sequence analysis is based on a subset of previously published 16S rRNA gene sequences from one of these studies (NCBI Sequence Read Archive accession SRP028774; Müller et al., 2014). Briefly, pyrosequencing of PCR-amplified 16S rRNA genes (V6–V9 region) was performed on DNA extracted from sediment slurry subsamples before and after 120 h of incubation, for one slurry per station. The subset of sequences that were significantly enriched after 120 h were clustered into OTUs to a ≥97% similarity threshold (Müller et al., 2014). A subset of 10 OTUs related to *Desulfotomaculum* that were enriched in our 10 stations was retained for further analysis (Supplementary Table S2). Here, we use the term “OTU,” equivalent to “phylotype” as used by Müller et al. (2014). We retain here the OTU naming scheme as defined in that study, with the prefix “TSP.” Prior to statistical analyses, the OTU by sample table was randomly subsampled to the smallest sample size (226 reads; Supplementary Table S2) one hundred times, and median Jaccard (presence-absence) and Bray-Curtis (read count) dissimilarity matrices were calculated using MacQIIME version 1.9.1 (Caporaso et al., 2010). Analyses were repeated on normalized raw data (standardized and square-root transformed OTU-by-sample read-count data) with little effect on the results (Supplementary Table S3). Cluster analysis was performed using Primer-E version 6.1.5 (Plymouth, United Kingdom).

### Oligotyping

Oligotyping was employed to identify subtle 16S rRNA gene nucleotide variation (termed “oligotypes”) within individual *Desulfotomaculum* OTUs from the above analysis. Oligotyping uses Shannon entropy to identify individual nucleotide positions representative of biologically relevant genetic variation among closely related sequences (Eren et al., 2013, 2016) while minimizing the potential influence of random sequencing error (Kleindienst et al., 2016). OTUs having greater than 100 total reads from at least one of the 10 stations were oligotyped individually using oligotyping version 1.7, following the recommendations by Eren et al. (2013) and described in Supplementary Information. To ensure even comparisons, stations having less than 10 reads for a given OTU were removed from the oligotyping analysis, hence the number of stations in the oligotyping analysis was often less than the number of stations in which that OTU was originally detected (Table 2). Inverse Simpson’s Index (Table 2) and rarefaction curves (Supplementary Figure S1) were calculated using MOTHUR version 1.33.0 (Schloss et al., 2009). Prior to statistical analyses (below), we followed a similar normalization approach as employed by Buttigieg and Ramette (2014). Specifically, for each OTU individually, any stations with fewer than 10 reads after oligotyping analysis were removed, and then oligotype-by-sample abundance tables were standardized by total number of reads per sample, square-root transformed, and converted into Bray-Curtis dissimilarity matrices.

**Table 2:** Summary of oligotyping analyses for each of six *Desulfotomaculum* OTUs, including oligotype richness, diversity, and GenBank blast hits.

OTUs	No. stations detected	No. stations used in oligotyping analysis (No. reads)	Alignment length (bp)	Entropy positions	No. oligotypes observed (raw no. before noise filtering^a^)	No. reads for most abundant oligotype (% of total)	Simpson’s Diversity Index (1/D)	Top GenBank blast hit for most abundant oligotype (% identity, % coverage)
TSP004	10	7 (2684)	351	58, 130, 138, 144	8 (14)	2343 (87.3)	1.30	JQ304695 *Desulfotomaculum* sp. Lac-2 (99.7, 94.3)
*TSP004^b^*		*6 (2308)*	*393*	*1, 34, 179, 186*	*6 (7)*	*2020 (87.5)*	*1.29*	*Same as above*
TSP006	7	4 (1819)	377	5, 7, 8, 146, 324, 334	10 (12)	829 (45.6)	3.34	JQ304697 *Desulfotomaculum* sp. For-1 (99.3, 94.3)
TSP015	9	4 (751)	277	41, 46, 52, 66	7 (9)	553 (73.6)	1.77	JQ741985 uncultured *Clostridiales* bacterium clone 22 (98.5, 100)
TSP032	2	1 (458)	352	9, 42, 280	5 (6)	291 (63.5)	2.18	FN396785 uncultured marine bacterium clone s5_8_I_31 (100, 93.3)
TSP045	2	2 (234)	284	70, 75	3 (4)	209 (89.3)	1.66	AY918123 *Desulfotomaculum salinum* strain 781 (95.4, 100)
TSP085	3	2 (890)	353	6, 38, 44	5 (5)	701 (78.8)	1.56	AY918123 *Desulfotomaculum salinum* strain 781 (95.4, 100)

*^*a*^To reduce noise, oligotypes consisting of three or fewer reads were eliminated from further analysis, as explained in the Section “Materials and Methods.” ^*b*^Oligotyping was repeated for TSP004 to allow for a longer alignment but inclusive of fewer total reads.*

### Statistical Analyses

**FIGURE 2:**
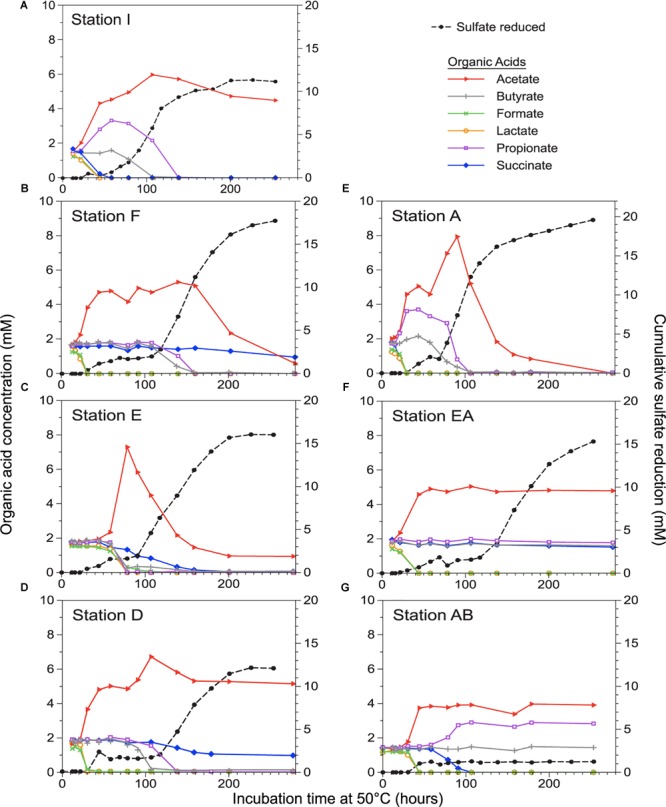
Progress in sulfate reduction (measured on the right axis) and organic acid concentrations (measured on the left axis) over time in sediment incubations for seven out of ten stations **(A–G)**, ordered north to south. Organic acid concentrations were not determined for stations CN, BE, and AH (not shown; see Supplementary Table S1).

To test for relationships between biotic similarity (community-wide traits, OTU composition, or oligotype composition) and geophysical variables (geographic distance or water depth), we performed simple Mantel-type tests with the RELATE function in Primer-E version 6.1.5 (Plymouth, United Kingdom) using weighted Spearman’s rank correlation coefficient (ρ) with significance at *p* ≤ 0.05. A geographic distance matrix was created using the open-source website “Geographic distance matrix generator” version 1.2.3 (Ersts, 2006) with WGS84 spheroid mode. For water depth, a Euclidean distance matrix was calculated on normalized data (also see SI). For organic acid trait data (Supplementary Table S1), a binary phenotype matrix (1 indicating consumption, 0 indicating no consumption) for acetate, butyrate, formate, lactate, and propionate in each of the 10 sampling stations was converted into a dissimilarity matrix using the Czekanowski metric (Legendre and Legendre, 1998). Succinate was not considered in this analysis as its dynamics were not well-coupled to sulfate reduction, but rather it appeared to be consumed via succinate decarboxylation to propionate. Consumption was defined as a clear and consistent decrease in concentration at any point over the course of incubation. In some cases, “consumption” is the net result of production followed by depletion (e.g., acetate in Figure 2B,C,E).

To test for relationships among number of oligotypes observed and number of reads, alignment length, or stations, Pearson correlations were performed in StatPlus for Mac version 5.9.33. To test for differences in overall genetic variation among stations, we performed Analysis of Molecular Variance (AMOVA) for each OTU separately in MOTHUR version 1.33.0 using the same sequence alignments as for oligotyping analysis. For AMOVA, we tested the null hypothesis that sequence reads from stations have the same level of nucleotide divergence as the entire dataset combined (sequences from all stations pooled together). We used the same alignments as used for oligotyping, such that the number of samples in each AMOVA analysis is equivalent to the number of stations in the oligotyping analyses (Table 2). At the oligotype level, AMOVA and RELATE tests were only performed for OTUs having greater than two stations in the oligotyping analysis.

### Availability of Data and Research Materials

The raw 16S rRNA gene amplicon sequence datasets analyzed for this study can be found in the NCBI Sequence Read Archive under accession number SRP028774, https://trace.ncbi.nlm.nih.gov/Traces/sra/?study=SRP028774 (Müller et al., 2014). The raw biogeochemical data, any other data files supporting the conclusions of this manuscript, and other materials such as protocols and analytical steps will be made available by the authors, without undue reservation, to any qualified researcher. Requests to access the datasets and other materials should be directed to the corresponding author.

## Results

### Organic Acid Resource Use by Enriched Endospore-Forming Thermophiles

Sulfate reduction was detected in sediments from all ten sampling stations (Figure 1 and Table 1) within 30 h of incubation at 50°C (Figure 2 and Supplementary Table S1), indicating the ubiquitous presence of viable, spore-forming thermophilic SRB along the west coast of Svalbard. Sulfate-reducing activity was in good correspondence with the consumption of different organic acids (Figure 2). Initial sulfate reduction was always associated with the consumption of formate and lactate and the production of acetate. Relatively small amounts of sulfate were consumed (3 mM or less) before this initial activity leveled off, consistent with sulfate reduction coupled to incomplete oxidation of 1 mM lactate and complete oxidation of 1 mM formate, as well as other substrates that may be derived from the sediment organic matter (Hubert et al., 2010). This resource use pattern was observed in all stations, suggesting thermophilic SRB with corresponding metabolic capabilities were ubiquitous across the study area.

Beyond this initial response, however, sulfate reduction and organic acid consumption patterns were not uniform among stations. For all sediments except two (stations AB and EA) a second distinct exponential increase in sulfate reduction, with concomitant changes in the concentration of the organic acids, was apparent near or after 60 h of incubation (Supplementary Table S1). In these instances, this second phase encompassed most of the overall sulfate reduction observed (Figure 2 and Supplementary Table S1). For most stations this second phase also exhibited corresponding consumption of butyrate and propionate, though the timing of consumption was not always clearly coupled to the onset of the second phase of sulfate reduction. In contrast, stations AB and EA lacked a second phase of sulfate reduction with concomitant butyrate and propionate consumption, suggesting that thermophilic SRBs capable of utilizing these two organic acids were possibly absent from these stations or if present, did not grow; e.g., due to the absence of other bacteria (see SI for further discussion). While acetate was always generated as a by-product of incomplete lactate oxidation during the initial phase of sulfate reduction, its subsequent depletion during the second phase was observed in only 3 out of 8 stations, suggesting patchy occurrence of thermophilic organisms capable of acetate utilization in the different fjord sediments sampled. Stations E and A were the only sediments for which all six measured organic acids were consumed. Despite overall spatial differences in organic acid turnover, there was no relationship between organic acid consumption traits and geographic distance (ρ = -0.269, *p* = 0.75) or water depth (ρ = 0.042, *p* = 0.31).

### Diversity and Biogeography of Enriched *Desulfotomaculum*

**FIGURE 3:**
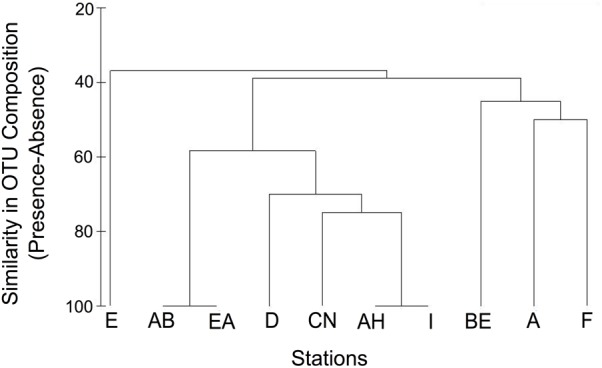
Similarity in *Desulfotomaculum* OTU composition among sampling stations based on presence-absence of 10 OTUs.

Spatial differences between fjords in organic acid turnover coupled to different phases of sulfate reduction coincided with the sequential appearance of different *Desulfotomaculum* spp. (a time-resolved example is shown in Supplementary Figure S3 and discussed in Supplementary Information). Therefore, we focused on sulfate-reducing *Desulfotomaculum* for subsequent 16S rRNA gene sequence analysis. We detected a total of ten enriched *Desulfotomaculum* OTUs (defined at ≥97% similarity; Supplementary Table S2) across all stations, with one OTU, TSP004, present in all stations. The most OTU-rich *Desulfotomaculum* communities were found at Stations BE and A, each having 7 OTUs, with 4 in common. Stations AB and EA were the least OTU-rich, sharing the same two *Desulfotomaculum* OTUs: TSP004 and TSP015. While not ubiquitous, TSP006 and TSP015 were still very common, occurring in 7 and 9 out of the 10 stations, respectively. In contrast, two other OTUs were detected in just one station each, both occurring in station BE (TSP036 and TSP072). While TSP004 was detected in all stations, the presence of the other 9 OTUs was much more variable (Supplementary Table S2). Despite these observations, there was no significant relationship between enriched *Desulfotomaculum* OTU composition and geographic distance or water depth (Figure 3 and Supplementary Table S3). For example, stations AH and I are among the geographically most distant pairs (approx. 220 km), yet are identical with respect to the occurrence pattern of *Desulfotomaculum* OTUs (Figure 3). In contrast, stations A and D were very different in terms of OTU occurrence (60% dissimilar; Figure 3) despite being one of the geographically closest pairs (approx. 23 km).

### Microdiversity Within *Desulfotomaculum* OTUs

**FIGURE 4:**
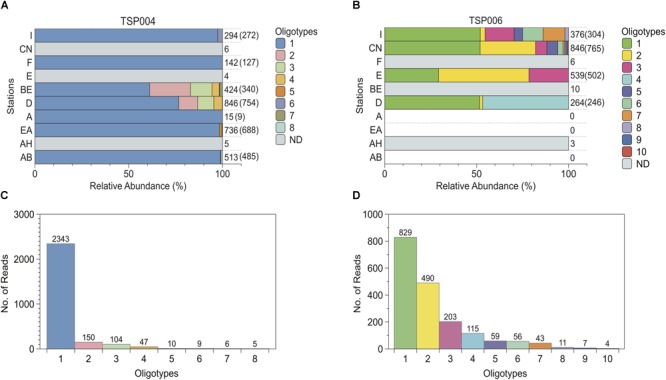
Distribution and relative abundances of oligotypes within *Desulfotomaculum* OTUs TSP004 **(A,C)** and TSP006 **(B,D)**. **(A,B)** Numbers to the right indicate the total number of reads detected in each station, followed in parentheses by the number of reads retained in the final oligotyping analysis. “ND”, gray bars = not determined, i.e., oligotyping not performed due to low numbers of reads. White/blank indicates stations in which the OTU was not detected in pyrosequencing libraries. Sampling stations are ordered in approximately northern-most to southern-most on the *y*-axis. **(C,D)** Rank-abundance showing number of sequence reads per observed oligotype. For all panels, different colors represent different oligotypes; oligotyping results based on the shorter alignment for TSP004 are shown.

We further investigated the diversity of enriched *Desulfotomaculum* spp. in our samples by using oligotyping to resolve fine-scale variation within OTUs (<3% nucleotide differences). Oligotype diversity varied substantially among OTUs (Table 2, Figure 4, and Supplementary Figure S2). TSP006 was more diverse than TSP004 (Supplementary Figure S1 and Table 2), even though TSP004 was the most abundant OTU overall and present in all stations. TSP004 mainly consisted of one highly predominant oligotype, TSP004 oligotype 1, which represented >87% of the reads for this OTU (Figure 4A,C). By contrast, TSP006 consisted of 10 oligotypes that were more even in overall relative abundance (Simpson’s Diversity Index = 3.34; Table 2), with the most abundant oligotype among these 10 representing less than 50% of the total diversity for this OTU (Figure 4B,D). Accordingly, the number of oligotypes for a given OTU was not significantly correlated with the number of reads used in oligotyping analysis (*R* = 0.66; *p* = 0.10), number of stations the OTU was detected in (*R* = 0.69, *p* = 0.13), or the alignment length (*R* = 0.41, *p* = 0.36). Therefore, the number of oligotypes per OTU does not seem to be affected by these methodological factors.

For all *Desulfotomaculum* OTUs, oligotype composition varied among stations (Figure 4 and Supplementary Figure S2) and within-OTU genetic variation differed significantly among stations (AMOVAs: F_s_ > 40 and *p* < 0.001 for all OTUs; Table 3). However, individual OTUs exhibited different patterns in the way oligotypes were distributed spatially. TSP004 was highly uniform among stations, with a single oligotype constituting nearly all of the reads in all but two of the seven stations included in its analysis (Figure 4A,C); and even in these two stations (BE and D) the predominant oligotype made up >60% of the reads. These two stations explain the overall weaker AMOVA results with fewer significant pairwise station comparisons for TSP004. Consequently, there was no relationship between oligotype composition and geographic distance or water depth for TSP004 within Svalbard (Table 3). The most abundant TSP004 oligotype (TSP004 oligotype 1) was 99.4% identical to oligotype 2 (i.e., 2 bp difference over 351 total bp) and all oligotypes had at least 98.9% identity (no more than 4 bp difference) (Figure 4A,C).

**Table 3:** Results for Analysis of Molecular Variance (AMOVA) and RELATE tests on oligotype composition for three *Desulfotomaculum* OTUs.

				Abundance (Bray-Curtis)				Presence-Absence (Jaccard)			
	AMOVA^a^			Geographic distance^b^		Water depth^b^		Geographic distance^b^		Water depth^b^	
	Fs	Global *p*-value	Pairwise station comparisons	ρ	*p*-value	ρ	*p*-value	ρ	*p*-value	ρ	*p*-value
TSP004	42.40	<0.001^∗∗^	14 out of 21, *p* < 0.05	–0.47	0.956	0.40	0.155	–0.17	0.703	0.45	0.070
*TSP004^c^*	*40.55*	*<0.001^∗∗^*	*13 out of 15, p < 0.05*	–*0.48*	*0.901*	–*0.14*	*0.539*	–*0.23*	*0.761*	*0.10*	*0.270*
TSP006	84.41	<0.001^∗∗^	All significant, *p* < 0.001	0.49	0.045^∗^	–0.62	0.954	0.91	0.088	–0.16	0.520
TSP015	172.11	<0.001^∗∗^	All significant, *p* < 0.001	0.68	0.170	0.32	0.172	0.96	0.058	0.71	0.045^∗^

*^*a*^AMOVAs were performed on the alignments used for oligotyping analysis. ^*b*^Results of Spearman rank correlation tests between similarity in oligotype composition and geographic distance or water depth. Significant relationships (*p* ≤ 0.05) are indicated by asterisks. ^*c*^Oligotyping was repeated on TSP004 to allow for a longer alignment but inclusive of fewer total reads (see Table 1).*

In contrast, TSP006 exhibited relatively more spatial variation in oligotype composition (Figure 4B). In fact, TSP006 oligotype composition within Svalbard was significantly correlated with spatial distance (RELATE test: ρ = 0.49, *p* = 0.045; Table 3). Two TSP006 oligotypes were detected in all four stations in the oligotyping analysis, but the other eight TSP006 oligotypes were highly variable in occurrence and relative abundance among stations, displaying a north to south trend (Figure 4B). For example, TSP006 oligotypes 3 and 6 were only detected in the northernmost stations. Further, unlike TSP004, all AMOVA *post hoc* pairwise station comparisons for TSP006 were significant. TSP006 oligotypes are 98.2–99.7% identical to each other (at most 6 and at minimum 1 bp difference over 377 total bp).

Oligotyping other OTUs also revealed spatial variation (Supplementary Figure S2). For example, for TSP015, no single oligotype was detected in all 4 analyzed stations. TSP045 consisted of just three oligotypes across two stations, with none being found in both stations. Finally, although TSP085 was dominated by a single oligotype, it also comprised several additional rare oligotypes appearing in just one station.

## Discussion

In accordance with our hypothesis, we found that enriched thermophilic endospores vary strikingly in their spatial distributions on the western coast of Svalbard with respect to all three metrics of diversity – organic acid-use traits at the community-level, OTU composition, and sub-OTU level microdiversity within the 16S rRNA gene. Patterns at all definitions of diversity displayed a general trend that some taxa are widespread while others have more limited distributions. One *Desulfotomaculum* OTU (TSP004) and two community-wide traits (formate and lactate consumption) were ubiquitously present at all stations. In contrast, other OTUs were more patchily distributed, being detected in as few as a single station to as many as nine stations. At the sub-OTU level, we also observed oligotypes that were more widely distributed across multiple stations and others that were geographically more restricted (Figure 4 and Supplementary Figure S2). Importantly, however, a relationship with geographic distance only emerged at the highest level of taxonomic resolution achieved with oligotyping. For instance, TSP006 oligotype composition was significantly correlated with geographic distance, even though there was no evidence for a correlation between geographic distance and overall OTU composition or organic acid-use traits. Community-wide organic acid-use traits generally mirrored the spatial variation detected at the OTU level, with neither being sufficient for delineating clear biogeographic patterns in enriched thermophilic communities.

The biogeographic patterns observed here for thermophilic endospores at the small scale of western Svalbard are best explained by historical factors associated with one or more allochthonous warm source environments where the thermophilic spore-forming populations were once active. As such, distribution patterns in Svalbard sediments can be used to speculate on the nature of these source environments. For example, since *Desulfotomaculum* OTUs vary in their presence-absence and in their diversity and distribution of oligotypes, there appear to be multiple sources or source environments that vary in their environmental conditions and/or spatial locations. In this way, historical factors other than (or in addition to) dispersal vary among taxa of thermophilic endospores and thus shape their contemporary distribution in cold sediments. This is one of the first studies to demonstrate that historical factors associated with a past environment drive the contemporary biogeographic distribution of a marine microbe in sediments.

These findings support previous suggestions that in other parts of the world, thermophilic endospores could be derived from multiple sources, including different marine deep biosphere environments (Hubert et al., 2009, 2010; de Rezende et al., 2013; Müller et al., 2014; Bell et al., 2018; Chakraborty et al., 2018). Potential marine deep biosphere sources include: (1) exposed crust and off-axis hydrothermal systems at the seafloor in areas flanking mid-ocean ridge spreading centers (Summit and Baross, 2001), where crustal-seawater fluid flow occurs at temperatures in the range of growth for diverse bacteria (Orcutt et al., 2011), including thermophilic *Desulfotomaculum* spp.; and (2) oil or gas reservoirs, which consist of deeply buried sediments at geothermally elevated temperatures. Reservoir environments are also capable of supporting thermophilic microbial communities (Head et al., 2003; Magot, 2005) and are connected to the overlying ocean by upward fluid flow at hydrocarbon seeps (Hubert and Judd, 2010). Indeed, *Desulfotomaculum* spp. have previously been identified in off-axis hydrothermal samples in other parts of the world (Brazelton et al., 2006; Cha et al., 2013; Müller et al., 2014; Robador et al., 2016) and in oil reservoir samples in the nearby North Sea (Rosnes et al., 1991; Nilsen et al., 1996) and elsewhere (Tardy-Jacquenod et al., 1998; Çetin et al., 2007; Chakraborty et al., 2018). These environments have unique geology that could hypothetically constrain the ecological and evolutionary history of their *in situ* microbial populations in different ways (Medlin, 2007). Here, we use these environments as examples to consider how differences at potential source habitats could equate to differences in past ecological and evolutionary pressures, and thus explain the observed patterns of diversity in Svalbard surface sediments. Then, we discuss alternative explanations.

For instance, TSP004 is ubiquitous along the west coast of Svalbard, consists of a single dominant oligotype, and lacks a relationship with geographic distance. Because a single oligotype of TSP004 is widely distributed across all stations, the source population is apparently not highly genetically differentiated with respect to the 16S rRNA gene at the regional scale investigated here. Therefore, we postulate that the majority of individuals of TSP004, representing the main dominant oligotype, have a common history [i.e., a common gene pool (Medlin, 2007)] that consists of origins in a spatially extensive, inter-connected, and well-mixed warm marine ecosystem that is far enough offshore to impact the entire north-south fjord system that we investigated. The nearby Arctic and northern Mid-Atlantic ridges are one set of possible sources that satisfy these criteria (Crane et al., 2001; Kelley et al., 2002; Edmonds et al., 2003), especially with recent discoveries of off-axis venting close to Svalbard (Pedersen et al., 2010; Jaeschke et al., 2014). At local to regional scales within warm ridge environments, relatively high rates of both dispersal and colonization by microbial individuals could occur through a constant exchange of crustal fluids and seawater (Elderfield and Schultz, 1996; Kelley et al., 2002; Hutnak et al., 2008; Fisher and Wheat, 2010; Orcutt et al., 2011; Jungbluth et al., 2016). This could result in a widespread, well-mixed *Desulfotomaculum* population that is relatively genetically homogenous with respect to the 16S rRNA gene (Slatkin, 1987; Cohan and Perry, 2007; Hartl and Clark, 2007), like the dominant oligotype we observe for TSP004. This perspective contrasts with other studies reporting an island-like habitat structure for hydrothermal submarine crustal environments and inhibited bacterial dispersal between individual venting mounds (Huber et al., 2010; Mino et al., 2013). However, we speculate that because *Desulfotomaculum* is spore-forming, it may more easily overcome these potential dispersal barriers compared to other thermophilic and hyperthermophilic residents.

In contrast, all other OTUs are much more patchily distributed with patchily distributed oligotypes. One explanation for this pattern is past circumstances that facilitated spatial isolation, allowing genetic divergence of different oligotypes. Such circumstances could have arisen in environments that are not as spatially connected as mid-ocean ridge systems. Subsurface petroleum reservoirs can be separated at depth within geologic formations, such that adjacent reservoirs even just a few kilometers apart can be physically isolated allowing divergent evolution in deep biosphere “islands” (Lewin et al., 2014). Reservoir fluids escaping to the surface at seabed hydrocarbon seeps could serve as patchily distributed localized point sources of closely related but non-identical taxa, like the oligotypes we have mapped for TSP006. In other words, microbial populations residing in reservoirs are relatively more closed to exchange with outside populations as compared to exposed axial ocean crustal fluid systems. In fact, the finding by Lewin et al. (2014) that similar taxa derived from adjacent but separated reservoirs share ca. 98% similarity across metagenomes agrees with the microdiversity distinguished within TSP006 (oligotypes are highly identical, 98.2–99.7% in the variable V6–V9 region). The pool of independent populations derived from multiple isolated reservoirs would be expected to have at least some genetic variation, resulting in a relatively patchy distribution once dispersed to overlying cold sediments especially if fine scale genetic analysis is employed. TSP006 is consistent with this as it consists of multiple genetically differentiated populations (corresponding to different oligotypes), many of which are patchily distributed, i.e., found in no more than a few stations (Figure 4B,D). Further, TSP006 is not present in all stations and exhibits a north-south decrease in compositional similarity (Figure 4B) which could be caused by dispersal-limitation from localized point source(s). The closest match to TSP006 oligotypes 1 and 2 in GenBank at 98% sequence identity is derived from a subsurface hydrocarbon contaminated aquifer (JQ086982; Tischer et al., 2013).

It is also possible that instead of multiple habitats or spatial isolation, different oligotypes and/or OTUs arose within the same source habitat where they coexist. In order for multiple, closely related populations to coexist, ecological theory suggests that those populations must be ecologically differentiated somehow. In such a scenario, TSP006, for example, could be derived from a single reservoir, or from a ridge flank environment, where different TSP006 oligotypes represent coexisting populations adapted to different ecological conditions or with different functional traits (as discussed in Larkin and Martiny, 2017). Indeed, this has been observed for other bacterial groups sampled directly from marine hydrothermal systems (Huber et al., 2007; Brazelton et al., 2010; Anderson et al., 2017). However, when TSP006 is detected, regardless of oligotype composition, it always emerges late in our enrichment incubation experiments and is associated with the removal of propionate and butyrate (SI and Supplementary Figure S3), suggesting that TSP006 oligotypes could share similar functions. Our study does not resolve whether and how coexisting oligotypes differ with respect to their physiology, ecology, or genomic content. Competing explanations of the historical factors determining TSP006 distributions highlight the ongoing need for further research on the causes and consequences of microbial microdiversity at the sub-OTU level.

Another explanation is that different *Desulfotomaculum* taxa and/or oligotypes have origins in other habitats, such as naturally geothermal or anthropogenic terrestrial sources. This has been suggested for *Desulfotomaculum* endospores enriched from an urban river estuary in the United Kingdom (Bell et al., 2018). We speculate that this may be the case for the distinct and minor TSP004 oligotypes that we detected uniquely within Isfjorden’s stations BE and D (Figure 4). Isfjorden is not only the largest fjord on the west coast of Svalbard but also the location of Longyearbyen, the capital and main settlement of Svalbard’s 2500 inhabitants. Isfjorden sediments may receive input related to human activities, e.g., mining and/or wastewater, which can be non-marine habitats for thermophilic SRB (Tasaki et al., 1991; Kaksonen et al., 2006; Widdel, 2006). Diversity at the broader 97% OTU-level also suggests the possibility of a localized source specifically influencing Isfjorden, as we also detected three OTUs (TSP032, TSP036, and TSP072) that were restricted to one or more of the four stations in Isfjorden. While acknowledging these possibilities, we speculate that most of the *Desulfotomaculum* taxa detected in this study are more likely derived from marine geothermal sources because (1) enrichment medium selects for sulfate-reducers capable of growing in brackish to saline conditions, (2) Svalbard experiences little, and only very local, human influence due to its sparse population, and (3) we have detected similar *Desulfotomaculum* taxa in isolated coastal fjords in northern Svalbard [Magdalenefjorden, station I, in this study, and even further north in Smeerenburgfjorden, in previous work (Hubert et al., 2009, 2010)], which have no human settlements.

The explanations above offer plausible past ecological scenarios that could have generated the observed patterns. These hypothesized origins and scenarios should be tested further, for example, by including samples directly from the proposed source environments. Most endospores are resistant to commonly used DNA extraction methods; thus, alternative, specialized methods like that tested by Wunderlin et al. (2013) may be necessary in order to capture DNA directly from endospores. Therefore, currently published sequence libraries from mid-ocean ridge and petroleum reservoir environments may not always include taxa highly related to those observed in our study. Additionally, many bacteria, including *Desulfotomaculum* spp., carry multiple copies of the 16S rRNA gene in their genomes (Větrovský and Baldrian, 2013), with both copy number and 16S sequence varying both among and within species. Therefore, it is possible that different oligotypes are derived from the same cell or strain. However, oligotypes were not detected in similar proportions across all samples, but are generally distributed very unequally (Figure 4 and Supplementary Figure S2) suggesting they represent distinct yet related organisms.

Overall, this study highlights how the ability to detect the role of historical factors on microbial biogeography requires more sensitive taxonomic resolution than standard 97% OTU definitions (Hanson et al., 2012; Chase and Martiny, 2018). Oligotyping analysis was able to reveal biogeographic variation within *Desulfotomaculum* OTUs at levels representing no more than 1.2% divergence across 351–377 bp within the V6–V9 region of the 16S rRNA gene. Recent studies have also uncovered distributional patterns in marine bacteria using oligotyping of 16S rRNA gene sequences. For example, oligotypes of sediment bacteria at an Arctic Long-Term Ecological Research site, roughly 100 km west of Svalbard, showed relationships with environmental variables that were not predictable by analyses based on 97% OTU community composition (Buttigieg and Ramette, 2014). In another study, the relative abundance of *Pelagibacter* oligotypes that were >99% identical to each other exhibited a strong, annually recurring seasonal pattern that was masked at the 97% OTU level (Eren et al., 2013). Similarly, oligotypes of aquatic *Vibrio* and estuarine *Synechococcus* demonstrated fine-scale differentiation according to environmental differences (Schmidt et al., 2014; Mackey et al., 2017, respectively). Building on these examples, our study has not only used oligotyping to unmask biogeographic structure, but to further reveal that such structure can be generated by differences in historical factors among and within OTUs. Thus, populations within OTU-level groupings do not necessarily have a common ecological and evolutionary history and higher resolution methods must be employed to tease apart the influence of history on contemporary microbial diversity. This probably at least partially explains the lack of agreement to-date on the relative importance of historical factors in shaping biogeographic patterns for microbes (Martiny et al., 2006; Hanson et al., 2012).

This work substantiates that historical factors associated with former environments are capable of generating ecologically relevant biogeographic patterns in spore-forming marine microbes. Even for dormant spores, everything is not everywhere, and individual populations can exhibit different distributional patterns as a result of divergent histories. High-resolution analysis of microdiversity within the 16S rRNA gene was necessary to detect contrasting historical signatures that could have been shaped by different conditions of potential deep biosphere source habitats. From this, we have developed further testable hypotheses on the ecological nature of potential former source habitats and how this could have influenced the biogeography of microdiverse lineages of thermophilic *Desulfotomaculum* in Arctic sediments. Because subsurface environments are difficult to sample directly and to reproduce artificially, these taxa may serve as more accessible indicators for (1) the presence of undiscovered subsurface environments and resources (Hubert and Judd, 2010) and (2) the ecological and evolutionary history of microbial life within those environments.

## Author Contributions

CAH, CRH, AM, AL, and BJ designed the study. CRH collected the samples. CRH, AM, CD, and RA performed the laboratory analysis. AM conducted the bioinformatics. CAH performed the bioinformatics (oligotyping) and analyzed the data. CAH wrote the manuscript with contributions from all of the co-authors.

## Conflict of Interest Statement

The authors declare that the research was conducted in the absence of any commercial or financial relationships that could be construed as a potential conflict of interest.
