# Supplementary material for: Historical Factors Associated With Past Environments Influence the Biogeography of Thermophilic Endospores in Arctic Marine Sediments
Source: Front Microbiol. 2019 Feb 28;10:245. doi: 10.3389/fmicb.2019.00245 (PMC6403435; doi:10.3389/fmicb.2019.00245)
Supplement: Supplementary file 1 [file Data_Sheet_1.pdf]

Supplementary Information

Historical factors associated with past environments influence the biogeography of thermophilic endospores in Arctic marine sediments

China A. Hanson, Albert L. Müller, Alexander Loy, Clelia Dona, Ramona Appel, Bo Barker Jørgensen, and Casey R. J. Hubert

Supplementary Materials and Methods

Oligotyping

For each OTU in the oligotyping analysis, the 10-15% shortest reads were removed from initial alignments and the remaining longer reads re-aligned using muscle in MacQIIME version 1.9.1. Uninformative gap characters were removed and alignments trimmed to the shortest read to eliminate terminal end gap characters. Alignments were then used as input for an initial round of oligotyping using the 3-4 highest entropy positions (defined as having >0.20 entropy value in the overall entropy analysis). To minimize the effect of potential homopolymer error, alignments near entropy peaks were manually inspected (Eren et al., 2000), and peaks within 1 bp of any homopolymeric region consisting of 4 or more repeats (e.g., AAAA or GGGGG) were not used for oligotyping. Note also that sequence libraries were previously denoised with PyroNoise as described in Müller et al (2014). To further reduce the influence of noise (i.e. variation caused by random sequencing error), we filtered oligotyping results using the parameter setting, A = 4; meaning that oligotypes having ≤3 reads across the whole dataset were omitted. We did however include oligotypes if they were present in only one station (parameter setting: s = 1). Resulting oligotypes having 15 or more reads and purity scores of at least 0.90 were considered fully resolved. Oligotypes that did not meet these criteria underwent a second round of oligotyping using the highest 1-2 entropy positions; no more than 2 rounds of oligotyping were required for any OTU. We did not attempt to resolve oligotypes having less than 15 reads in order to reduce the likelihood of losing sequence data due to low abundance.

Water depth

We included water depth as a geophysical variable in our analyses in addition to geographic distance for two main reasons. First, water depth may serve as a proxy variable for thermospore dispersal via sedimentation and/or for particular point sources. For example, thermospores derived from habitat sources located further off-shore and/or in deeper waters may be more detectable in sediments from deeper waters and less detectable in shallow near-shore sediments. Secondly, other work in the Arctic at the nearby HAUSGARTEN LTER reported significant depth-related changes in benthic microbial communities both at the OTU-level (Jacob et al., 2013) and at the oligotype-level within OTUs (Buttigieg and Ramette, 2014). The authors were unable to definitively interpret these results and suggested that future work should consider the influence of water depth on benthic microbial biogeography. There was no evidence for a correlation between water depth and geographic distance (RELATE test: ρ=-0.023, p=0.174); hence our use of simple Mantel tests using the RELATE function when testing for relationships between biotic similarity and geophysical variables.

Denaturing Gradient Gel Electrophoreis (DGGE)

Prior to DGGE, DNA was extracted from incubation subsamples as described in the Materials and Methods. Two-step nested PCRs were performed using the *Desulfotomaculum*-specific 16S rRNA

gene primers, DEM116F and DEM1164R (Stubner and Meuser, 2000), followed by 341f-gc/907r (universal bacterial 16S rRNA primers). Identity of specific bands was determined by gel extraction followed by Sanger sequencing (Fig. S3).

Supplementary Results and Discussion

Organic acid consumption coupled to SR

We included a trait-based measure in order to represent community variation that may not be apparent from 16S rRNA gene sequences alone, since expression of traits may vary independently of 16S rRNA genes (Gevers et al., 2005). Further, it is unclear whether trait- or taxonomic-based metrics are better suited for studying microbial biogeography (Green et al., 2008). Our method to assess traits - growth upon heating pre-pasteurized environmental samples under anaerobic conditions (i.e. enrichment incubations) - elicits a measurable whole-community response. This response includes sulfate reduction coupled to organic acid consumption as well as organic acid production and consumption from the net result of all thermophilic microbes present in the samples that are capable of germination and activity under these enrichment conditions. For this reason, our study was not designed to link organic acid use traits to taxonomic identity.

Despite this, we did observe a sequential pattern of organic acid resource consumption well-coupled to sulfate reduction that can be explained by the successional enrichment of particular *Desulfotomaculum* OTUs over time in sediment incubations (Fig. S3). The time-resolved analysis presented in Fig. S3 shows that TSP004 germinates quickly and appears to be responsible for the initial increase in SR coupled to consumption of lactate and formate. This OTU and set of traits were detected in all samples. The leveling off of this early SR suggests that TSP004 growth becomes limited by the depletion of these electron donors.

This first phase of SR is often followed by a second phase of SR and the enrichment of other OTUs, notably TSP006. When TSP006 is present, it is usually, but perhaps not always, responsible for the second phase in SR coupled to consumption of propionate and butyrate. Evidence for this comes from the fact that stations EA and AB lack both a TSP006 rRNA signal (in both 16S sequence libraries and the DGGE analysis (Fig. S3)), and both lack propionate and butyrate consumption (Fig. 2). In the one station where TSP006 was not detected, but propionate and butyrate were consumed (station A; Table S2 and Fig. 2), a different OTU is instead probably responsible (TSP085, TSP032, or both). Further studies on isolated representatives of these *Desulfotomaculum* OTUs would be needed in order to rule out the possibility that these organic acid consumption patterns are the result of several *Desulfotomaculum* OTUs acting in concert and/or with non-sulfate-reducing thermophiles (Volpi et al., 2017). In the present study, the organic acid use patterns provided a framework and hypotheses for assessing biogeographic resolution using complementary genetic approaches.

In addition to enrichment of different *Desulfotomaculum* spp (Fig. S3), marine sediment incubations also yield fermentative thermophiles (e.g. different *Clostridiaceae*), which can influence organic acid dynamics (Müller et al., 2014; Volpi et al., 2017) and thus the growth of SRB. Activity of thermophilic fermenters likely explains succinate removal in 4 of the 7 sediments for which organic acids were measured.

References

Buttigieg PL, Ramette A. 2014. Biogeographic patterns of bacterial microdiversity in Arctic deep-sea sediments (HAUSGARTEN, Fram Strait). Front Microbiol 5.

Eren AM, Borisy GG, Huse SM, Welch JLM. 2014. Oligotyping analysis of the human oral microbiome. Proc Natl Acad Sci USA 111:E2875-E2884.

Gevers D, Cohan FM, Lawrence JG, Spratt BG, Coenye T, Feil EJ, Stackebrandt E, Van de Peer Y, Vandamme P, Thompson FL, Swings J. 2005. Re-evaluating prokaryotic species. Nat Rev Microbiol 3:733-739.

Green JL, Bohannan BJ, Whitaker RJ. 2008. Microbial biogeography: from taxonomy to traits. Science 320:1039-43.

Jacob M, Soltwedel T, Boetius A, Ramette A. 2013. Biogeography of deep-sea benthic bacteria at regional scale (LTER HAUSGARTEN, Fram Strait, Arctic). PLOS One 8.

Müller AL, de Rezende JR, Hubert CR, Kjeldsen KU, Lagkouvardos I, Berry D, Jørgensen BB, Loy A. 2014. Endospores of thermophilic bacteria as tracers of microbial dispersal by ocean currents. ISME J 8:1153-1165.

Stubner S and Meuser K. 2000. Detection of *Desulfotomaculum* in an Italian rice paddy soil by 16S ribosomal nucleic acid analyses. FEMS Microbiol Ecol 34:73-80. doi:10.1111/j.1574-6941.2000.tb00756.x

Volpi M, Lomstein BA, Sichert A, Roy H, Jorgensen BB, Kjeldsen KU. 2017. Identity, abundance, and reactivation kinetics of thermophilic fermentative endospores in cold marine sediment and seawater. Front Microbiol 8:131.

Table S1. Summary of sulfate reduction, organic acid use traits, and presence of TSP004 and TSP006 in pyrosequencing libraries for each station.

Station	Sulfate Reduction Phase 2[a]	Formate consumption	Lactate consumption	Succinate consumption	Proprionate consumption	Butyrate consumption	Acetate consumption	Cumulative Sulfate Reduced[b] (mM)	TSP004 detected	TSP006 detected
**I**	yes	yes	yes	yes	yes	yes	no	11.6	yes	yes
**CN**	yes	ND[c]	ND	ND	ND	ND	ND	13.9	yes	yes
**F**	yes	yes	yes	no	yes	yes	yes	18.3	yes	yes
**E**	yes	yes	yes	yes	yes	yes	yes	16.0	yes	yes
**BE**	yes	ND	ND	ND	ND	ND	ND	15.4	yes	yes
**D**	yes	yes	yes	no	yes	yes	no	11.3	yes	yes
**A**	yes	yes	yes	yes	yes	yes	yes	19.6	yes	no
**EA**	no	yes	yes	no	no	no	no	15.3	yes	no
**AH**	yes	ND	ND	ND	ND	ND	ND	5.28	yes	yes
**AB**	no	yes	yes	yes	no	no	no	1.24	yes	no

[a] presence of a second increase in sulfate reduction detectable no sooner than 60 hours of incubation
[b] Total concentration of sulfate removed after 253 hours of incubation
[c] ND = not determined

Table S2. OTU table showing the number of pyrosequencing reads per *Desulfotomaculum* OTU per sampling station; and the total number of reads by OTU, by station, and combined.

	Desulfotomaculum OTUs											
**Station**	**TSP004**	**TSP006**	**TSP015**	**TSP046**	**TSP085**	**TSP032**	**TSP036**	**TSP045**	**TSP072**	**TSP119**	**Total No. Reads by Station**	**Total No. OTUs by Station**
**I**	294	376	3	0	0	0	0	0	0	0	673	3
**CN**	6	846	24	0	0	0	0	252	0	0	1128	4
**F**	142	6	3	4	0	0	0	0	0	71	226	5
**E**	4	539	0	0	1055	0	0	0	0	0	1598	3
**BE**	424	10	8	83	6	0	1	0	74	0	606	7
**D**	846	264	60	0	0	1	0	0	0	0	1171	4
**A**	15	0	1	3	54	500	0	9	0	3	585	7
**EA**	736	0	18	0	0	0	0	0	0	0	754	2
**AH**	5	3	703	0	0	0	0	0	0	0	711	3
**AB**	513	0	20	0	0	0	0	0	0	0	533	2
**Total # reads**	2985	2044	840	90	1115	501	1	261	74	74	7985	

Table S3. Results of RELATE tests for correlations between similarity in *Desulfotomaculum* OTU composition and geographic distance or water depth.

	Rarefied to minimum sample[a]				Standardised and transformed raw read count data[b]			
	Geographic Distance		Water Depth		Geographic Distance		Water Depth	
OTU Similarity Metric	**ρ**	*p*-value	**ρ**	*p*-value	**ρ**	*p*-value	**ρ**	*p*-value
presence-absence (Jaccard)	-0.26	ns[c]	-0.17	ns	-0.26	ns	-0.12	ns
abundance (Bray-Curtis)	0.03	ns	-0.23	ns	-0.10	ns	-0.24	ns

[a] OTU-by-sample read count tables were randomly subsampled to the minimum sample size one-hundred times, prior to similarity matrix calculation
[b] OTU-by-sample read count tables were standardized by the total numbers of reads per sample and then square-root transformed, prior to similarity matrix calculation
[c] ns = not significant; $p>0.24$ in all cases

Supplementary Figures

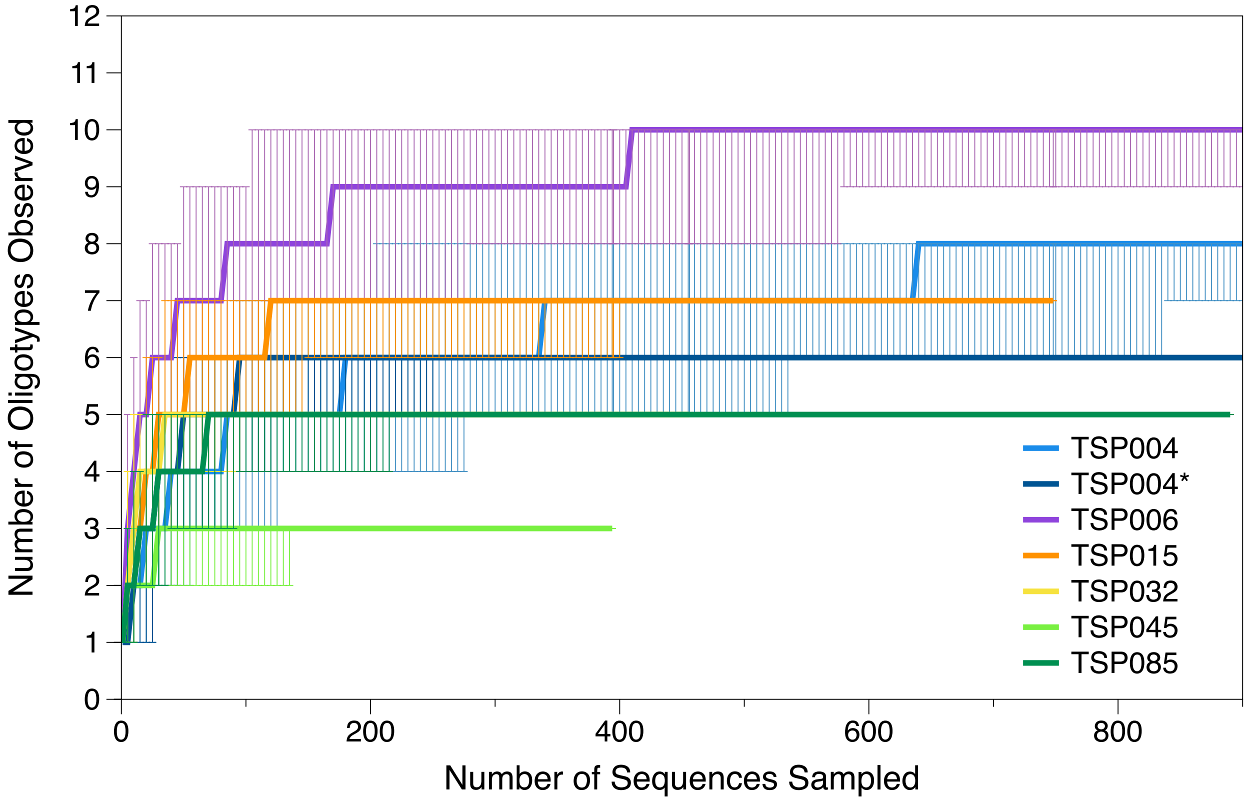

Fig. S1. Rarefaction curves for number of oligotypes observed for each of six *Desulfotomaculum* OTUs. * = Oligotyping was repeated for TSP004 to allow for a longer alignment but inclusive of fewer total reads (see Methods and Table 2 in the main text). Error bars represent 95% confidence intervals. Note that the y-axis has been truncated to 900 for clarity, as number of oligotypes observed did not increase beyond this.

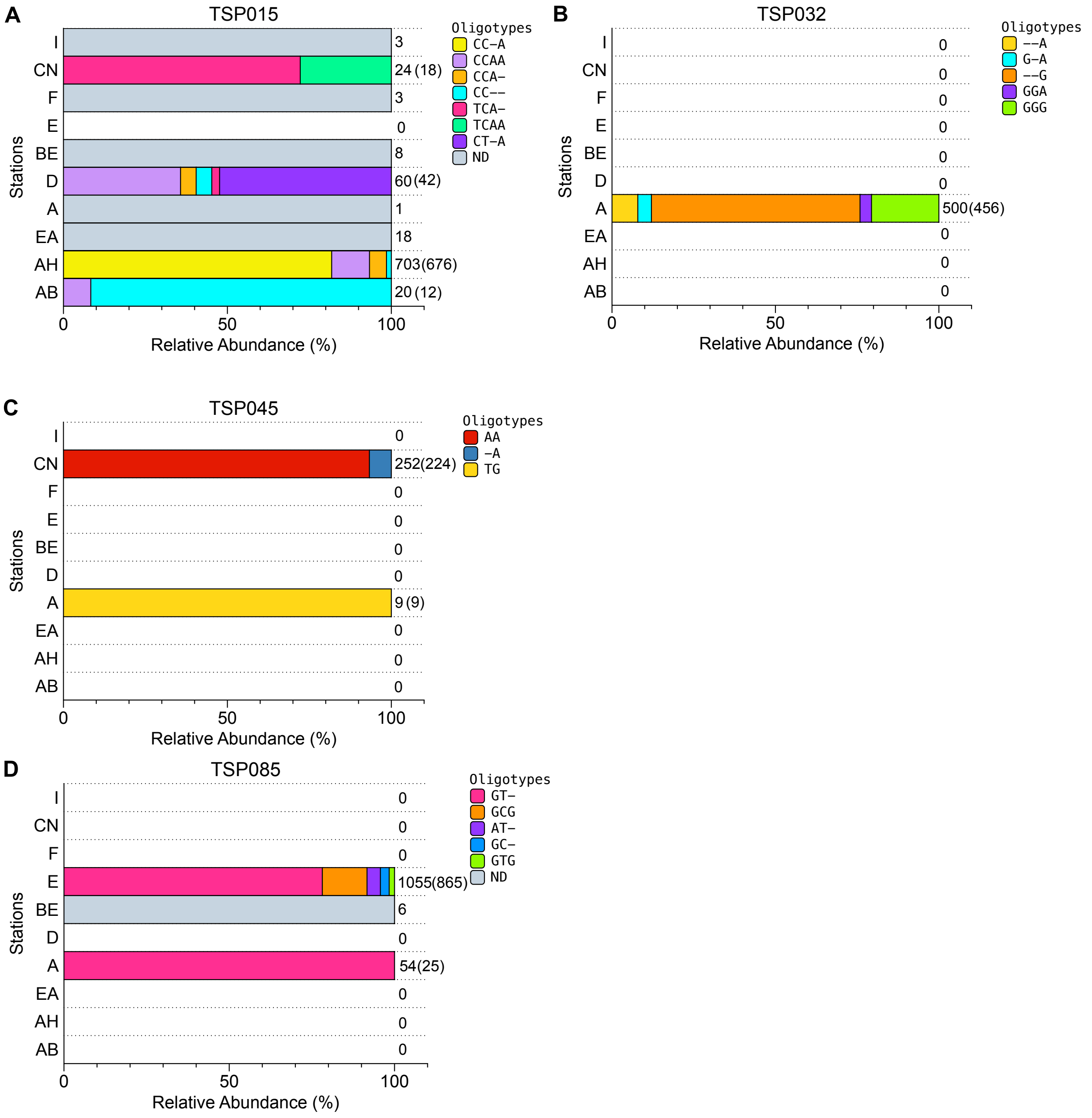

Fig. S2. Distribution and relative abundances of oligotypes within four other *Desulfotomaculum* OTUs. Numbers to the right indicate the total number of reads detected in each station, followed in parentheses by the number of reads retained in the final oligotyping analysis. "ND", grey bars = not determined, i.e. oligotyping not performed due to low numbers of reads. White/blank indicates stations in which the OTU was not detected in pyrosequencing libraries. Sampling stations are ordered in approximately northern-most to southern-most on the y-axis. Within a panel, different colours represent different oligotypes.

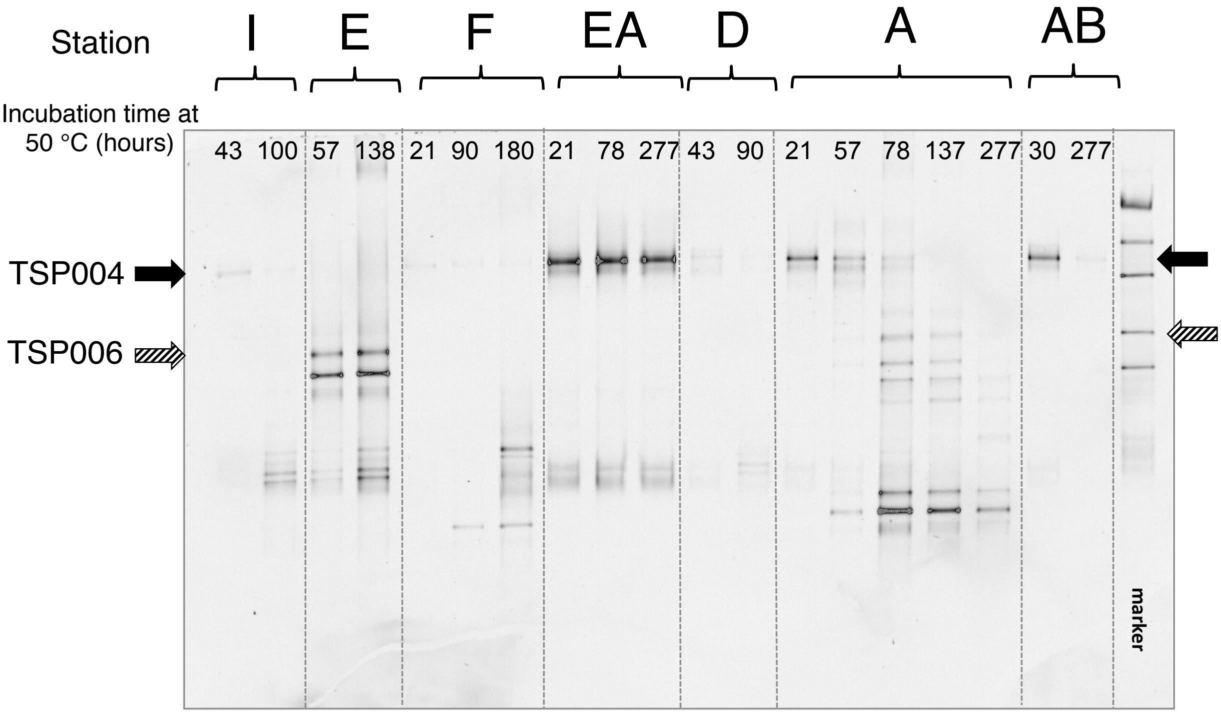

Fig. S3. Denaturing Gradient Gel Electrophoresis (DGGE) image showing the diversity of enriched *Desulfotomaculum* over time in sediment incubation experiments for selected stations. This image demonstrates 1) the existence of spatial differences in *Desulfotomaculum* composition and 2) a successional pattern of bands over time during the course of incubations (i.e., the band corresponding to TSP004 becomes weaker over time in some cases). We assume that different bands represent *Desulfotomaculum* diversity at the approximate species level, and therefore make no inference about different oligotypes here. Solid arrows indicate the approximate gel migration position for TSP004 amplicons. Hatched arrows indicate the approximate gel migration position for TSP006 amplicons.
